# Donor chimerism and immune reconstitution following haploidentical transplantation in sickle cell disease

**DOI:** 10.3389/fimmu.2022.1055497

**Published:** 2022-12-09

**Authors:** Yaya Chu, Julie-An Talano, Lee Ann Baxter-Lowe, James W. Verbsky, Erin Morris, Harshini Mahanti, Janet Ayello, Carolyn Keever-Taylor, Bryon Johnson, Rona S. Weinberg, Qiuhu Shi, Theodore B. Moore, Sandra Fabricatore, Brenda Grossman, Carmella van de Ven, Shalini Shenoy, Mitchell S. Cairo

**Affiliations:** ^1^ Department of Pediatrics, New York Medical College, Valhalla, NY, United States; ^2^ Department of Pediatrics, Hematology/Oncology and BMT, Children’s Hospital of Wisconsin, Medical College of Wisconsin, Milwaukee, WI, United States; ^3^ Department of Pathology, Children’s Hospital of Los Angeles, University of Southern California, Los Angeles, CA, United States; ^4^ Department of Pathology, New York Medical College, Valhalla, NY, United States; ^5^ Department of Medicine, Division of Hematology and Oncology, Medical College of Wisconsin, Milwaukee, WI, United States; ^6^ New York Blood Center, New York, NY, United States; ^7^ Department of Epidemiology and Community Health, New York Medical College, Valhalla, NY, United States; ^8^ Department of Pediatrics, David Geffen School of Medicine at University of California, Los Angeles, Los Angeles, CA, United States; ^9^ Department of Pathology and Immunology, Washington University, St Louis, MO, United States; ^10^ Department of Pediatrics and Transfusion Medicine, Washington University, St Louis, MO, United States; ^11^ Department of Medicine, New York Medical College, Valhalla, NY, United States; ^12^ Department of Microbiology and Immunology, New York Medical College, Valhalla, NY, United States; ^13^ Department of Cell Biology, New York Medical College, Valhalla, NY, United States; ^14^ Department of Anatomy, New York Medical College, Valhalla, NY, United States

**Keywords:** sickle cell disease, donor chimerism, immune reconstitution, HLA antibodies, CD34 enrichment, donor grafts, NK, haploidentical transplantation

## Abstract

**Introduction:**

We previously reported the initial results of a phase II multicenter transplant trial using haploidentical parental donors for children and aolescents with high-risk sickle cell disease achieving excellent survival with exceptionally low rates of graft-versus-host disease and resolution of sickle cell disease symptoms. To investigate human leukocyte antigen (HLA) sensitization, graft characteristics, donor chimerism, and immune reconstitution in these recipients.

**Methods:**

CD34 cells were enriched using the CliniMACS^®^ system with a target dose of 10 x 10^6^ CD34^+^ cells/kg with a peripheral blood mononuclear cell (PBMNC) addback dose of 2x10^5^ CD3/kg in the final product. Pre-transplant HLA antibodies were characterized. Donor chimerism was monitored 1-24 months post-transplant. Comprehensive assessment of immune reconstitution included lymphocyte subsets, plasma cytokines, complement levels, anti-viral T-cell responses, activation markers, and cytokine production. Infections were monitored.

**Results:**

HLA antibodies were detected in 7 of 11 (64%) evaluable patients but rarely were against donor antigens. Myeloid engraftment was rapid (100%) at a median of 9 days. At 30 days, donor chimerism was 93-99% and natural killer cell levels were restored. By 60 days, CD19 B cells were normal. CD8 and CD4 T-cells levels were normal by 279 and 365 days, respectively. Activated CD4 and CD8 T-cells were elevated at 100-365 days post-transplant while naïve cells remained below baseline. Tregs were elevated at 100-270 days post-transplant, returning to baseline levels at one year. At one year, C3 and C4 levels were above baseline and CH50 levels were near baseline. At one year, cytokine levels were not significantly different from baseline.

**Discussion:**

These results suggest that haploidentical transplantation with CD34-enriched cells and peripheral blood mononuclear cell addback results in rapid engraftment, sustained donor chimerism and broad-based immune reconstitution.

## Introduction

Sickle cell disease (SCD) is an inherited hemoglobinopathy affecting an estimated 100,000 patients in the US and 220,000 worldwide (1–3). A point mutation in the β-globin gene contributes to increased hemoglobin S polymerization which plays a major role in the complications of SCD (4–7). Investigation of gene correction utilizing autologous hematopoietic stem cell transplantation (HSCT) has been promising, but challenges remain including maintaining therapeutic levels of beta globin gene expression and preventing insertional mutagenesis (8, 9).

For SCD patients, HSCT from human leukocyte antigen (HLA) matched sibling donors has resulted in ≥90% event free survival (EFS) with a low incidence of engraftment failure and transplant-related mortality (10–19). However, only 14-18% of patients with SCD have an unaffected HLA matched sibling donor (20, 21). Attempts to use unrelated donors have been associated with an unacceptably high rate of chronic GVHD (cGVHD) (22). Furthermore, many SCD patients are African Americans who have low likelihood of identifying an HLA matched unrelated donor, likely due to extraordinary HLA diversity in this population and/or rates of donor availability (23). Use of unrelated cord blood HSCT has been reported by our group and others, but this approach has resulted in high rates of engraftment failure and disease-free survival ranging from 50-78% (23–25). Additionally, recent reports for patients with SCD have found that mortality and graft failure rates were increased after alternative donor transplantation suggesting that HLA disparity is deleterious in this patient population (13, 26). T cell depletion methods have been employed to reduce AGVHD such as CD3/CD19 cell depletion (27), αβ T-cell/CD19 cell depletion (28), CD34 positive selection (29).

The need for prior red blood cell (RBC) transfusions in the treatment of patients with SCD often induces the development of HLA antibodies, a major risk factor for graft failure. A recent study of 114 pediatric SCD patients showed that despite leukoreduction, 33% of patients receiving RBCs developed donor-specific HLA antibodies (DSA) (30), which have been associated with graft rejection in HLA mismatched transplants (31–36).

HLA matched donor HSCT is a successful therapy for SCD. A recent study shows that HLA-haploidentical bone marrow transplantation with post-transplantation cyclophosphamide and thiotepa improves donor engraftment without significantly increasing morbidity or mortality in SCD patients (37). We sought to increase the use of this therapy by utilizing HLA haploidentical related donors. CD34 cells were enriched using the CliniMACS^®^ system with a target dose of 10 x 10^6^ CD34^+^ cells/kg with a PBMNC addback dose of 2x10^5^ CD3/kg in the final product (38). Our single-arm, open-label, prospective phase II, multicenter trial using myeloimmunoablative conditioning and haploidentical stem cell transplantation (HISCT) from haploidentical family members for patients with high-risk SCD demonstrated feasibility, safety, and excellent (90%) 1-year EFS with no evidence of graft failure (38). Here, we report an exceptionally low incidence of graft versus host disease, characteristics of pre-transplant HLA antibodies, details of engraftment, sustained donor chimerism, and broad-based immune reconstitution in this patient cohort.

## Materials and methods

### Ethics

This multi-site phase II study was approved by the institutional review board at each participating site prior to enrollment. Each patient and/or guardian signed a written informed assent/consent. This study was registered at clinicaltrials.org (NCT02675959).

### Study design and participants

Recipients (2.0 to 20.99 years) with homozygous SCD, with one or more high-risk features, adequate organ function and no HLA matched unaffected sibling donor were eligible as previously described (**Supplementary Figures 1**, **2**) (38). HLA matching was determined using intermediate resolution HLA-A and -B typing and high resolution HLA-DRB1 typing (11, 25, 39). Twenty-one eligible SCD patients (2-<21 yrs) were enrolled. Nineteen patients received hydroxyurea and azathioprine from day -59 to day -11, fludarabine day -17 -to -13, busulfan day -12 to -9; thiotepa day -8, cyclophosphamide on days -7 to -4, rabbit anti-thymocyte globulin day -5 to -2, and total lymphocyte radiation on day -2, as we have previously described (38). Donors were mobilized with filgrastim (15 mcg/kg/day) divided twice a day (bid) x eight doses prior to peripheral blood stem cell (PBSC) collection. A target of 10 × 10^6^ CD34 cells/kg of recipient body weight and MNC (2 × 10^5^ CD3 cells/kg of recipient body weight) was cryopreserved on day −59 prior to the start of myeloimmunoablative conditioning. The CD34^+^ enrichment was performed using a CD34^+^ reagent system (CliniMACS; Miltenyi Biotec). Mononuclear cells (2 × 10^5^ CD3 cells/kg of recipient body weight) were removed from the leukapheresis collection prior to CD34^+^ enrichment and were cryopreserved as a source of MNC addback (T cells) as previously described (38).

### Incidence of pre-existing HLA antibodies

Anti-HLA IgG antibodies were detected using LabScreen™ Single Antigen assays (One Lambda Inc., West Hills CA). High-risk antibodies were defined as mean fluorescence intensity (MFI) >5000 (31, 40). Low risk HLA antibodies were defined as 1000-5000 MFI. DSA were determined by comparing specificities of each patient’s high-risk HLA antibodies with the HLA-A, -B, and DRB1 types of their donors.

### Whole blood RBC donor chimerism

DNA was isolated from whole blood and a fraction of the blood enriched for erythroid lineage cells using CD71 microparticles from Miltenyi Biotech (described as RBC in the text). Chimerism was determined using a method for detecting short-tandem repeat units in genomic DNA as described previously (41). Each post-transplant sample was tested in duplicate or triplicate unless sample was limiting.

### Immunophenotyping

Immunophenoptyping of patient and donor PBMNCs was performed using antibodies against CD3, CD4, CD8, CD56, CD19, lineage markers (CD3, CD14, CD16, CD19, CD20 and CD56), CD27, HLA-DR, CD123, and CD11c (BD Biosciences, San Jose, California, USA). CD45RA (Beckman Coulter Inc. Brea CA, USA) was used to detect naïve T-cells and CD127 (BD Biosciences, San Jose, California, USA) was used to detect memory B cells. CD4, CD25, CD127 and Foxp3 (BD Biosciences, San Jose, California, USA) were used to identify regulatory T-cells (Treg) cells.

### Th1/Th2 cytokine assays

Serum samples, obtained pre-transplantation and one year after HSCT, were stored at −80 °C. Concentrations of Th1/Th2 cytokines were measured using the Bio-Plex Pro Human 17 Cytokines Assay (Bio-Rad Laboratories, USA). IL-18 concentrations were measured using Enzyme-linked immunosorbent assays (ELISA) (Medical & Biological Laboratories Co, USA) as we previously described (42). For Bio-Plex Pro Human 17 Cytokines assays, the serum samples were analyzed according to the manufacturer’s instructions. In brief, 50 µl aliquot of sample was diluted 1:4 with sample diluent, incubated with antibody-coupled beads, washed, incubated with biotinylated secondary antibodies, and finally incubated with streptavidin-phycoerythrin. The beads were read on a Bio-Plex® 200 instrument (Bio-Rad Laboratories, USA). The standard curves were generated with a dynamic range between 5 and 20,000 pg/ml, and the data were analyzed using Bioplex Manager Software.

IL-18 concentrations were analyzed in Enzyme-linked immunosorbent assays (ELISA) (Medical & Biological Laboratories Co.) according to the manufacturer’s instructions. Recombinant IL-18 standards were run as 1:2.5 serial dilutions. Serum samples were diluted 1:4 with assay diluent. 100ul of diluted samples and standard were added to microwells simultaneously and incubated for 1hrs at room temperature. Peroxidase conjugated anti-human IL-18 antibody was used and incubated for 1 hr at room temperature. After washing, ELISA plates were developed with 100ul substrate reagents (TMB/H_2_O_2_). TMB Stop Solution was added to halt the reaction. The absorbance at 450 nm was measured on a Molecular Devices Multifilter F5 plate reader (Molecular Devices, USA).

### Anti-viral T-cell assays

Overlapping peptides (15 amino acids in length) spanning the common immunodominant antigens from cytomegalovirus (pp65), adenovirus (hexon), and Epstein Barr virus (BZLF1) were added to PBMNC according to the manufacturer’s recommendations, generally at 1 µg/mL, and incubated for 6 h in the presence of 1 µg/ml of co-stimulatory monoclonal antibodies against CD28 and CD49d (BD Biosciences, Immunocytometry systems, San Jose, CA, USA) and Brefeldin A at a concentration of 10 µg/ml (Sigma, St Louis, MO, USA).

The peptide-stimulated cells were processed for intracellular Interferon (IFN)-γ staining. After fixing and permeabilizing the peptide-stimulated cells, the samples were stained using the premixed antibody cocktails to include: IFN-γ/CD69/CD4/CD3 (BD Fastimmune, Cat#337184), a second with IFN-γ/CD69/CD8/CD3 (BD Fastimmune, Cat# 346048), and the third to contain appropriate isotype controls. Negative controls included co-stimulatory antibodies and Brefeldin A (Sigma, Cat# 6542-5mg, final concentration 10µg/ml) at the same concentration without peptides. The positive control was Streptococcus Enterotoxin B (SEB; 1.5 µg/ml).

The cells were analyzed on a FACSCanto II flow cytometer using appropriate calibration and compensation beads. The analysis included an initial gate on CD3^+^ events (including dim cells), followed by gating on the appropriate subset (CD4^+^ or CD8^+^), and then gating on the subset co-expressing CD69 and IFN-γ. Data are expressed as the percentage of CD3^+^CD4^+^ or CD3^+^CD8^+^ cells producing IFN-γ in response to the peptide used for stimulation. The mean ± SEM of %IFN-γ^+^ in CD4 cells and in CD8 cells in the non-pulsed cells are 0.09% ± 0.02% and 0.29% ± 0.06%, respectively. The positive response is defined as %IFN-γ^+^ in CD4 cells ≥ 0.09%, %IFN-γ^+^ in CD8 cells ≥ 0.29%.

### Natural killer cell receptor expression

Activating and inhibitory receptors on NK cells were assessed by flow cytometry as we have previously described^39^ using antibodies for CD56 dim and bright subsets, NK activating receptors, inhibitory KIRs, and C-type lectin receptors (NKp44, NKp46, CD158a, CD158b, KIR2DS, NKG2D, NKG2C, NKG2A and CD94 and CD94/NKG2D) (BD Biosciences, San Jose, California, USA). Cells were analyzed using a MACSQuant Analyzer (Miltenyi Biotec, Auburn, CA, USA).

### *In vitro* cytotoxicity and intracellular granzyme B and CD107a assays for NK cells

*In-vitro* cytotoxicity was assessed using K562 tumor targets at a 10:1 E:T ratio as previously described (43). Intracellular granzyme B and CD107a expression was measured by flow cytometry as previously described (43). Briefly, PBMCs were mixed with RPMI1640 medium or target cells at a ratio of 10:1 in the absence of exogenous cytokines in medium. For CD107a expression, anti-CD107a-FITC (Miltenyi Biotech) was added to each well and incubated for 1 hour at 37°C. After 1 hour, brefeldin A (Golgi Plug; BD Biosciences) was added to each well, and the cells were incubated for additional 3 hours. Cells were then washed, fixed, permeabilized using Cytofix/Cytoperm reagent kit (BD Biosciences), and resuspended in staining buffer containing anti-CD56-PE-Vio770 (Miltenyi Biotech). For Granzyme B levels, cells were incubated for 4 hours, washed, fixed, permeabilized using a Cytofix/Cytoperm reagent kit (BD Biosciences), and resuspended in staining buffer containing anti-CD56-PE-Vio770 (Miltenyi Biotech) and granzyme B-FITC (Miltenyi Biotech).

### Immunoglobulin and complement levels

IgM, IgG, and IgA levels and CH50, C3 and C4 levels were determined by clinical laboratories supporting each transplant site.

### Statistics

Mean ± standard error of the mean (SEM) was determined using GraphPad Prism 5 software (GraphPad Software, La Jolla, CA USA). Longitudinal data (e.g. cytokines, IgA/G/M levels, NK receptor expression) were analyzed using the mixed effect model in SAS 9.4 or in GraphPad Prism 5 software. Statistical significance was set at P<0.05. The cumulative incidence for GVHD was determined using the product limit method of Kaplan and Meier (44).

## Results

### Demographics

Nineteen recipients (mean+SEM age: 13 ± 1.2 yrs; age range: 3-20; 12 males, 7 females) received grafts from 18 parental haploidentical donors. Demographics, clinical characteristics, platelet and neutrophil engraftment, donor chimerism, and status at last follow-up are provided in **Table 1**.

**Table 1:** Clinical characteristics and clinical outcome results of HISCT recipients (N = 19).

Pt ID	Age yr	(M/F)	HLA Match Out of 6	Primary Risk Factor	Neurological Status at HISCT	Neutrophil engraftment Day	Platelet engraftment Day	Status (6.30.2022) Day
**526-001**	10	M	3/6	TCD	WNL	13	33	A/3531
**526-002**	13	F	3/6	Stroke	WNL	9	16	A/3418
**526-003**	20	F	4/6	Stroke	WNL	9	19	A/3124
**526-004**	18	F	3/6	ACS	WNL	10	NE	D/59
**526-005**	20	M	3/6	VOC	WNL	9	12	A/2914
**526-006**	12	M	3/6	VOC	WNL	9	44	A/2894
**526-007**	8	M	3/6	Silent infarct	WNL	11	21	A/2845
**526-008**	9	F	3/6	Stroke	WNL	9	17	A/2740
**526-009**	15	M	3/6	Stroke	WNL	6	8	A/2558
**526-010**	4	M	3/6	ACS	WNL	10	15	A/2610
**526-011**	17	F	3/6	Silent infarct	WNL	9	NE	D/141
**526-012**	14	M	3/6	TCD	WNL	9	90	A/2512
**526-013**	12	F	3/6	Stroke	WNL	9	14	D/390
**526-014**	20	F	3/6	Stroke	WNL	9	18	A/2439
**526-015**	10	M	3/6	ACS	WNL	10	33	A/2257
**526-016**	20	M	3/6	Stroke	WNL	10	19	A/2075
**526-018**	3	M	3/6	ACS	WNL	10	16	A/2078
**526-019**	11	M	3/6	Silent infarct	WNL	10	33	A/1809
**526-020**	11	M	3/6	Silent infarct	WNL	9	8	A/1802
**Summary mean ± SEM**	13 ± 1.2	12/7 (M/F)	3/6 N=18 4/6 N=1	N/A	N/A	9.5 ± 0.3	24.5 ± 4.7	med f/u 2610 days (59-3531)

HISCT, haploidentical stem cell transplantation; N, number; Pt ID, patient identifier; yr, year; M/F, male/female; HLA, human leukocyte antigen; TCD, transcranial Doppler abnormal; A, alive; ACS, acute chest syndrome; NE. not engrafted; D, death (004, VOC, 011, aGVHD, 013, cGVHD); VOC, veno-occlusive disease; N/A, not applicable; SEM, standard error of the mean; f/u, follow up; WNL, within normal limits.

### Donor grafts

**Figure 1:**
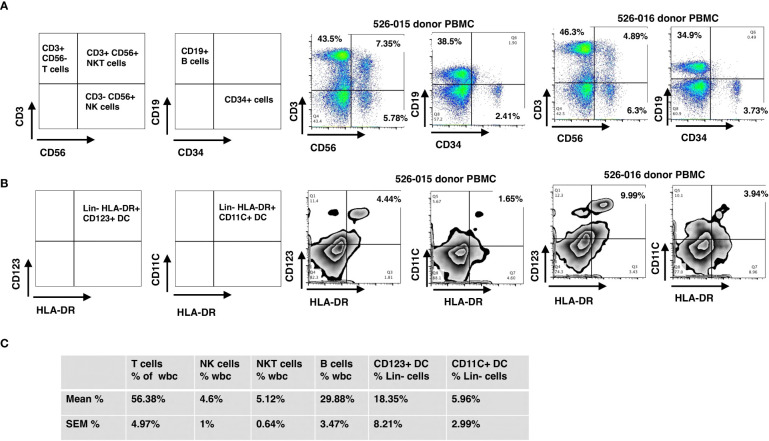
Immunophenotyping of mononuclear cells added back using flow cytometry. The final products for HSC grafts were characterized by staining with antibodies against: CD3, CD56, CD19, lineage markers (CD3, CD14, CD16, CD19, CD20 and CD56), HLA-DR, CD123, and CD11c. **(A)** Representative flow cytometry dot plots of T, NK, NKT and B cells (donor 526-015) of WBC. **(B)** Representative flow cytometry dot plots of CD123^+^ DC and CD11c^+^ DC (donor 526-015) of Lin- (lineage negative) cells. **(C)** Mean and SEM for the cell subsets of all donors. SEM, standard error of the mean.

Briefly, CD34 cell enrichment, purity and viability were 66 ± 3.4%, 97 ± 0.4% and 96.3 ± 0.7%, respectively, with 4.8 ± 0.1 log depletion of CD3^+^ cells. Grafts contained 10.9 ± 0.4 CD34-enriched PBSC/kg with a MNC addback containing 2.0x10^5^ CD3 cells/kg along with other immune cells including NK, NKT, B and dendritic cells. Since 97% of the cells were CD34^+^ after enrichment, the majority of immune cells infused in the final donor product were derived from the peripheral blood MNC addback. We measured immune cell content in the MNC addback of five grafts, which consisted of 56.4 ± 5.0% CD3 T-cells, 29.9 ± 3.5% CD19 B-cells, 4.6 ± 1% CD3-/CD56 NK cells, 5.1 ± 0.6% CD3^+^/CD56^+^ NK T-cells, 5.96 ± 2.99% CD11c+/HLA DR+ DC1 Lin- (lineage-negative) cells, and 18.35 ± 8.21% CD123^+^/HLA-DR+ DC2 Lin- cells (**Figures 1A–C**). The probability of 6-month overall survival (OS) was 94.7%, of 1-year OS was 89.5% and of 2-year OS was 84.2%, respectively (**Supplementary Figure 2**) (38).

### Characterization of pre-transplant HLA antibodies

By day -59 of conditioning, most patients (79%) had received multiple donor RBC transfusions. HLA antibodies were detected in 7 of 11 (64%) evaluable patients (**Table 2**). Only one patient had evidence of DSA at levels previously associated with graft rejection (31). This patient engrafted (day 10 for neutrophils, day 33 for platelets, 99% donor chimerism at one year).

**Table 2:** RBC transfusions history and HLA antibodies prior to HISCT (11 patients tested for HLA antibodies/19 total subjects).

Pt ID	HLA Match	Primary Risk Factor	RBC Transfusions	HLA Ab Day	Class I HLA Ab High-Risk	Class II HLA Ab High-Risk	Class I HLA Ab Low-Risk	Class II HLA Ab Low-Risk	Possible DSA
**526-005**	3/6	VOC	>10	D 0	None	None	A2	None	None Detected
**526-006**	3/6	VOC	6	Pre HSCT	None	None	None	None	None Detected
**526-007**	3/6	Silent infarct	11	Pre HSCT	None	None	None	None	None Detected
**526-008**	3/6	Stroke	21	Pre HSCT	None	None	None	None	None Detected
**526-009**	3/6	Stroke	28	Pre HSCT	None	None	None	None	None Detected
**526-011**	3/6	Silent infarct	35	Pre HSCT	None	None	A34	DR53	NE
**526-012**	3/6	TCD	>10	D 0	None	None	A2,A68,A3,A11,A24,B76, B45,B44	DR4,DQ7,DQ8	NE
**526-013**	3/6	Stroke	>10	D 0	None	None	None B57, B58, B7, B63, B81, B76, B45, B44, B27, B13	None DP19	NE
**526-014**	3/6	Stroke	28	D 0	None	None	C17	DQ5	NE
**526-015**	3/6	ACS	19	Pre HSCT	B7, B81, B13, B60, B61, B48, B27, B55, B56, B13, B51, B31, B8, B52, B57	None	B73, B82, B54, B38, B59, B78, B71, B63, B47, B62, B49, B18, B50, B46, B58, B76, B72, B75, B53, B35	None	B51
**526-016**	3/6	Stroke	0	D 0	A80, A2, B51, B78	None	A24, A68, A69, B35, B53, B57, B52, B58, B8, B18	None	None Detected

Pt ID, patient identifier; RBC, red blood cells; HLA, human leukocyte antigen; HISCT, haploidentical stem cell transplantation; DSA, donor-specific HLA antibodies; TCD, transcranial Doppler; ACS, acute chest syndrome; VOC, veno-occlusive disease; HSCT, hematopoietic stem cell transplantation; NE, not evaluable.

### Engraftment, donor chimerism, acute (Grade II-IV) and chronic GVHD

**Figure 2:**
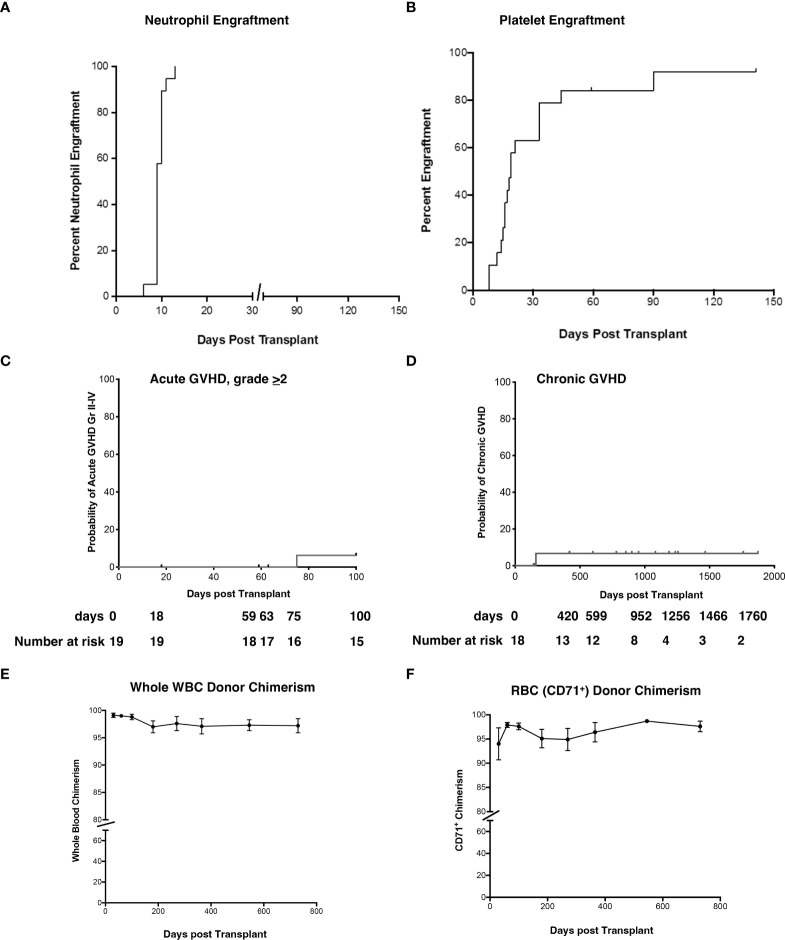
Neutrophil and platelet engraftment, cumulative incidence of Grade II-IV aGVHD and cGVHD, and whole blood and RBC donor chimerism. **(A, B)** The probability of neutrophil and platelet engraftment, **(C)** cumulative incidence of aGVHD, **(D)** cGVHD, **(E)** donor chimerism in whole blood, and **(F)** RBC (erythrocyte enriched fraction) were determined using the product limit method of Kaplan and Meier. Donor chimerism (% donor) was determined at 30d, 60d, 3-month, 6-month, 1 yr, and 2 yr post-HSCT. Panel E is peripheral blood and panel F represents RBC. Results are shown as the mean ± SEM. aGVHD, acute graft versus host disease; RBC, red blood cells; cGVHD, chronic graft versus host disease; HSCT, hematopoietic stem cell transplantation; SEM, standard error of the mean.

The median time to neutrophil and platelet engraftment was 9 and 19 days (**Figures 2A, B**), respectively, and the cumulative incidence of Grade II-IV acute GVHD (aGVHD) and extensive cGVHD was 6.2% and 6.7%, respectively (**Figures 2C, D**). Robust donor chimerism was observed by 30 days after HSCT (**Figures 2E, F**). One-year post-transplant, donor chimerism was 97.1 ± 1.4% in whole blood and 96.4 ± 2.0% in the RBC lineage (**Figures 2E, F**).

### Immune reconstitution

**Figure 3:**
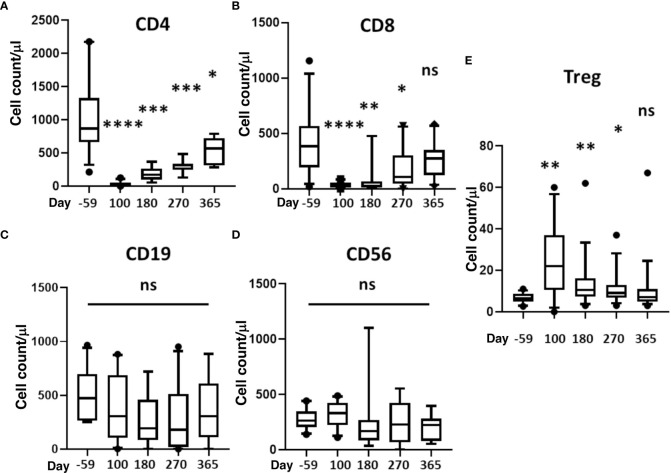
Reconstitution of T, B, NK, and Treg cells. Absolute cell counts were determined for **(A)** CD4^+^ T-cells, **(B)** CD8^+^ T-cells, **(C)** B cells, **(D)** NK cells (CD3^-^/CD56^+^), and **(E)** Treg (CD4^+^CD127^dim^CD25^hi^ Foxp3^+^). Measurements were made at baseline (day -59) and 100, 180, 270, and 365 days after HSCT. Statistically significant differences between baseline and post-HSCT timepoints are indicated as **** P<0.0001, *** P<0.005, ** P<0.001, * P<0.05. Differences that are not significant are labeled, ns. NK, natural killer; Treg, regulatory T cells; HSCT, hematopoietic stem cell transplantation; ns, not significant.

Reconstitution of CD4^+^, CD8^+^, CD19^+^, CD56^+^ and Treg cells is depicted in **Figures 3A–E**. It took about a year for CD4 and CD8 counts to return to pre-transplant levels (**Figures 3A, B**). There was no difference in B or NK cells counts (**Figures 3C, D**), and Treg (FoxP3+ CD4 T-cells) significantly increased at day 100 and remained at increased levels through day 270 before returning to host baseline levels at 1yr (**Figure 3E**).

**Figure 4:**
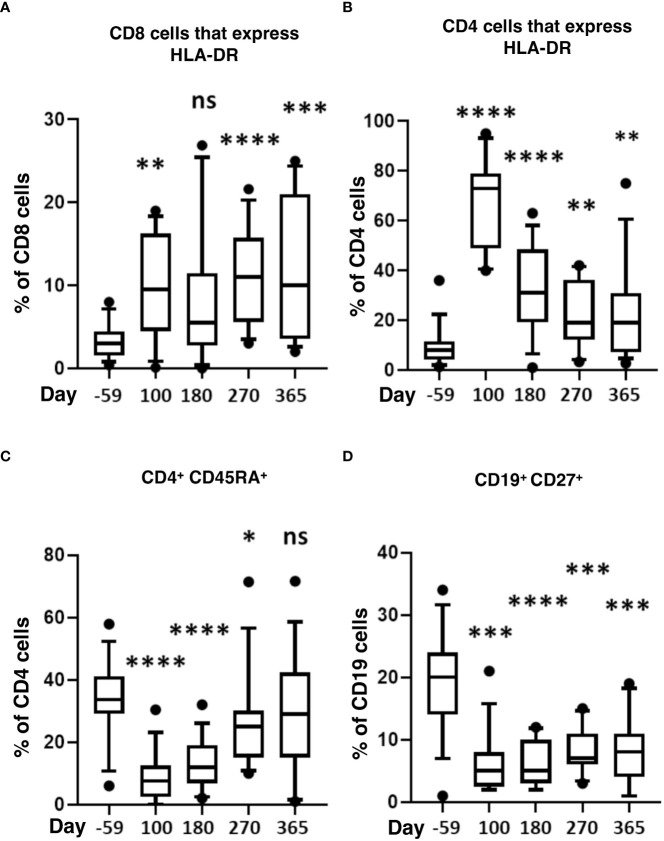
Markers of T-cell activation and memory after HISCT. The percentage of HLA-DR^+^ in CD8 **(A)**, the percentage of HLA-DR^+^ in CD4 **(B)**, and the percentage of CD45RA^+^ in CD4 T cells **(C)** is shown at baseline and indicated time points after HISCT. **(D)** the percentage of CD27^+^ in CD19^+^ B cells shown at baseline and indicated time points. Statistically significant differences between baseline and post-HSCT timepoints are indicated as **** P<0.0001, *** P<0.005, ** P<0.001, * P<0.05. ns, not significant; HSCT, hematopoietic stem cell transplantation; HLA, human leucocyte antigen; HISCT, haploidentical stem cell transplantation.

We also measured markers of immune activation and memory (**Figure 4**). During the first-year post-transplant, activated CD4 and CD8 T-cells significantly increased compared to pre-HSCT baselines (**Figures 4A, B**). CD4 naïve T-cells (CD45RA^+^) significantly decreased from day 100 to 270 and returned to normal by day 365 (**Figure 4C**). Compared to pre-transplant, memory B-cells (CD127^+^) were significantly lower throughout the post-HSCT period (day 100 to 365) (**Figure 4D**).

**Figure 5:**
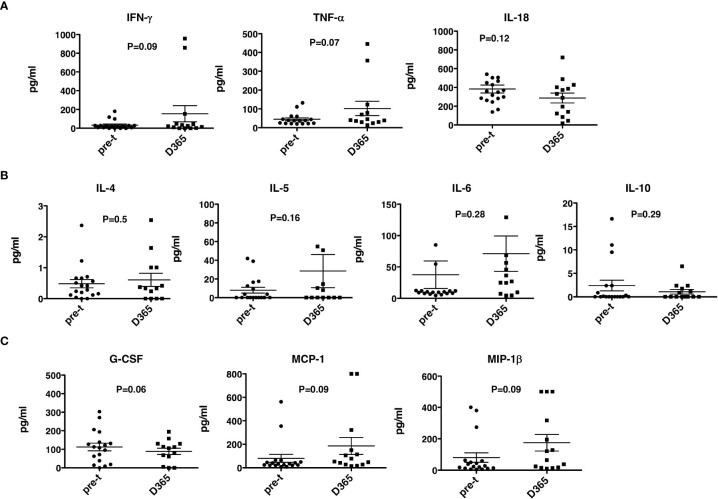
Th1 and Th2 cytokines and growth factors. Th1, Th2 cytokines and growth factor levels were determined in plasma before and 365 days after HSCT. **(A)** Th1 cytokines IFN-γ, TNF-α and IL-18 levels. **(B)** Th2 cytokines IL-4, IL-5, IL-6, and IL-10 levels. **(C)** G-CSF, MCP-1 and MIP-1β. HSCT, haploidentical stem cell transplant; ELISA, enzyme-linked immunosorbent assay; INF, interferon; TNF, tumor necrosis factor; GCSG, granulocyte colony stimulating factor. pre-t, pre-transplant; D, day.

**Figure 6:**
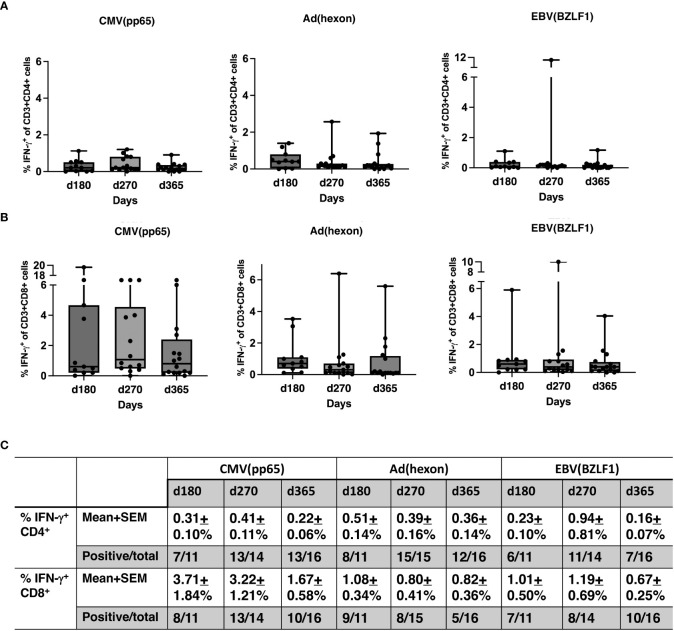
T-cell responses to viral antigens. **(A)** Percentage of IFN-γ^+^ CD4 cells in response to CMV (pp65), ADV (hexon), and EBV (BZLF1) shown in box and whisker plots. **(B)** Percentage IFN-γ^+^ CD8 cells in response to CMV (pp65), ADV (hexon), and EBV (BZLF1) shown in box and whisker plots. **(C)** Summaries of the % IFN-γ^+^ of CD4 cells (mean ± SEM), the % IFN-γ^+^ of CD8 cells (mean ± SEM), and the numbers of positive responses in response to CMV(pp65), ADV (hexon), and EBV (BZLF1) at day 180, day 270 and day 365. PBMNC, peripheral blood mononuclear cells; n, number; INF, interferon; CMV, cytomegalovirus; ADV, adenovirus; EBV, Epstein-Barr virus.

No significant differences were observed between pre- and post-HSCT plasma levels of IFN-γ, TNF-a, IL-4, IL-5, IL-6, IL-10, IL-18, MCP-1, MIP1b, and G-CSF (**Figures 5A–C**). For 17 recipients, CD3^+^/CD4^+^ and CD3/CD8^+^ T-cell responses (intracellular IFN-γ expression) to exogenous peptide pools of cytomegalovirus (CMV) (pp65), ADV (hexon) and Epstein-Barr virus (EBV) (BZLF1) were determined. T-cell responses (% IFN-γ^+^ in CD4 cells ≥ 0.09%, % IFN-γ^+^ in CD8 cells ≥ 0.29%) were observed from day +180 - +365 post-HISCT to all of the viral peptide pools (**Figures 6A, B**). The % IFN-γ^+^ of CD4 cells (mean ± SEM), the % IFN-γ^+^ of CD8 cells (mean ± SEM), and the numbers of positive responses in response to CMV(pp65), ADV (hexon), and EBV (BZLF1) at day 180, day 270 and day 365 are summarized in (**Figure 6C**).

### Infections

The incidence of viral reactivation was CMV = 5, EBV = 5, HHV6 = 3, and ADV = 2. Three fungal infections occurred (Candida = 2, Aspergillus fumigatus = 1). There were 10 bacterial infections; Enterobacter cloacae = 4, Bacillus = 2, Staph coag neg = 2, Elizabethkingia menignoseptica = 1, and Strep slavarius = 1. There were no direct infection-related deaths.

### NK cell reconstitution, receptor expression and function

**Figure 7:**
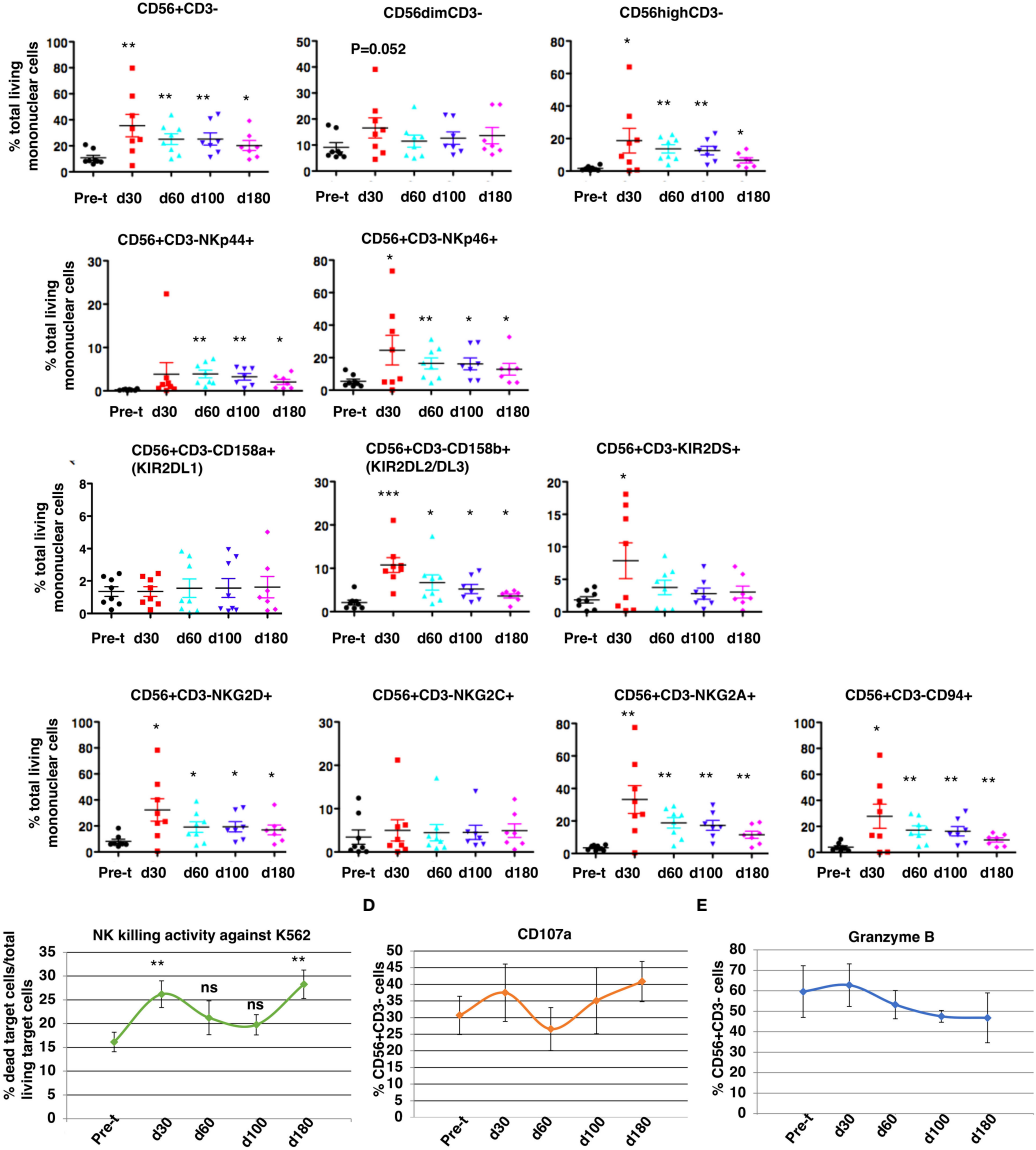
NK cell reconstitution. NK CD3^-^/CD56^+^cell reconstitution **(A)**, receptor expression **(B)**, NK cytotoxicity against K562 at a E:T=10:1 **(C)**, functional activation (CD107a) **(D)**, and protease expression (Granzyme B) **(E)** were characterized in the patients’ peripheral blood from 30 to 180 days after HSCT. Pre-t, pre-transplant; d, day; *p<0.05; **p<0.01; ***p<0.001; ns, not significant; NK, natural killer; HSCT, hematopoietic stem cell transplantation.

At 30 to 180 days after HSCT, NK cell subsets were measured by flow cytometry (**Figure 7**): CD3^-^/CD56^+^ cell reconstitution, NK receptor expression, functional activation (CD107a), and K562 cytotoxicity. NK cell (CD56dim/CD3^-^ and CD56bright/CD3^-^) reconstitution was extremely rapid and occurred as early as day 30 (d30: 35 ± 8.6% of total MNC, 95% CI = 15%, 56%, N = 8 vs pre-transplant: 11 ± 2.0% total MNC, 95% CI = 6%,15%, N = 8, P<0.01) and remained at normal levels through day 180, with robust levels of both cytotoxic CD56dim/CD3- NK cells and cytokine producing CD56 bright/CD3^-^ NK cells at day 30 (**Figure 7A**; P ≤ 0.05). The ratio of CD56 bright/CD3- NK cells to CD56dim/CD3- NK cells was about 1.13 at day 30. After HISCT, subsets of NK cells that expressed activating NK receptors (NKp44 and NKp46) were significantly higher than baseline (**Figure 7B**, top panels). Subsets of NK cells that expressed NK KIR receptors, KIR2DL2/DL3 and KIR2DS were also significantly higher post-transplant (**Figure 7B**, middle panels). Compared to baseline, the levels of activating C-type lectin-like receptor NKG2D and the inhibitory receptor NKG2A were significantly increased 30 days after HSCT (**Figure 7B**, bottom panels). CD94 levels were also significantly enhanced at day 30 (P<0.05) as compared to pre-transplant (pre-t) (**Figure 7B**, bottom panels). NKG2C levels were not significantly different before and after HISCT (**Figure 7B**, bottom panels).

When PBMNC from recipients were used to assay NK cell cytotoxicity against K562 (E:T=10:1), killing was significantly increased over pre-t baseline at 30 and 180 days after HSCT (**Figure 7C**). For gated NK cells, NK activation markers CD107a (**Figure 7D**) and granzyme B (**Figure 7E**) initially peaked 30 days after HISCT. There were no significant differences in absolute numbers of NK cell subsets that expressed NKp44, NKp46, CD158a, CD158b, KIR2DS, NKG2D, NKG2C, NKG2A and CD94 (**Supplementary Figure 3**).

### Immunoglobulin levels

**Figure 8:**
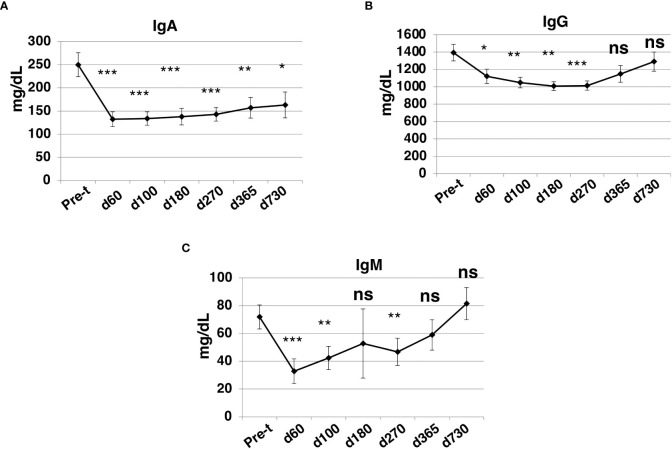
Immunoglobulin levels after HISCT. IgA **(A)**, IgG **(B)**, and IgM **(C)** levels in patients’ blood were determined by standard operation procedures of the clinical laboratories. Pre-t, pre-transplant; d, day; *p<0.05; **p<0.01; ***p<0.001; ns, not significant.

At day +60 after HISCT, IgA, IgG, and IgM levels were significantly lower than baseline levels (P<0.001, P<0.05, P<0.001, respectively) (**Figures 8A–C**). IgG and IgM levels returned to pre-HISCT levels, which were at normal ranges by one year, and levels were maintained two years post-transplant (**Figures 8B, C**). IgA levels remained below baseline levels (**Figure 8A**).

### Complement (CH50, C3 and C4)

**Figure 9:**
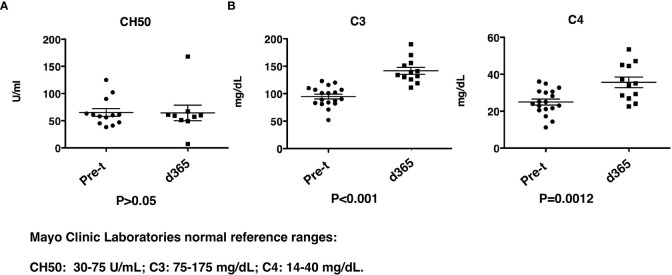
Complement levels after HISCT. **(A)** CH50 levels are shown at pre-transplantation and at day 365. **(B)** C3 and C4 levels are shown at pre-transplantation and at day 365. Pre-t, pre-transplant; d, day; HISCT, haploidentical stem cell transplantation.

CH50, C3 and C4 complement levels were determined before and 1yr after HSCT. While the total CH50 levels were similar before and post-transplant (**Figure 9A**), there was a significant increase (P<0.001) in both C3 and C4 levels (C3: 94.8 ± 4.3mg/dL vs 141.6 ± 6.4 mg/dL, and C4: 25.0 ± 1.6 mg/dL vs 35.6 ± 2.9 mg/dL) (**Figure 9B**). The levels of C3 and C4 before and post-transplant are both in the reported normal ranges even though there is an increase post-transplant.

## Discussion

In our multicenter trial of myeloimmunoablative conditioning and HISCT from haploidentical parental members for patients with high-risk SCD, we previously reported that overall survival at 1yr and 2yr were 90% and 84%, respectively (38). In this study, we reported characteristics underlying these successful transplants including 100% engraftment with rapid myeloid recovery (median 9 days), sustained donor whole blood and RBC chimerism, and broad-based rapid immune reconstitution, despite a high incidence of pre-existing HLA antibodies.

DSA have been reported to cause rejection of HLA-mismatched grafts and this is particularly relevant to SCD patients who often receive transfusions (45). In this study, consistent with prior reports from others, 64% of evaluable patients had anti-HLA antibodies before transplant (38). Fortuitously, only one of these patients had DSA detected at levels that have been reported to be strongly associated with rejection (31). This patient showed excellent engraftment (day 10 for neutrophils and day 33 for platelet engraftment, 99% donor chimerism at one year). Gaziev (46) reported detecting HLA antibodies in 6/18 (33%) hemoglobinopathy patients who were tested for HLA antibodies. Although 5 of the 6 patients experienced rejection, the role of HLA antibodies in these rejections is unclear because three patients who had no detectable HLA antibodies experienced rejection and only three of the five patients with HLA antibodies had donor-specific antibody levels that have been associated with rejection (46). Gaziev et al. speculated that graft content may play an important role in promoting engraftment in T-cell–depleted haplo-HCT in patients with hemoglobinopathies (46). Our study incorporated total lymphoid irradiation, and this may account for the absence of rejection, however, a larger study with patients who have clinically significant DSA is necessary to determine if this protocol reduces graft rejection when DSA antibodies are present.

Engraftment was robust in our trial with the median time to myeloid and platelet engraftment of 9 and 19 days, respectively. In contrast, protocols using PTCy reported slower engraftment with a median time to neutrophil engraftment of 26 (range 21–31) days (47). Additionally, de la Fuente et al. reported that the median time to neutrophil and platelet engraftment was 22 days and 28 days, respectively in SCD patients following haplo-BMT with post-transplantation cyclophosphamide and thiotepa treatment, which is significantly delayed compared to our approach (37). Bolanos-Meade et al. using a T-replete haploidentical post-cyclophosphamide approach had a time for neutrophil and platelet engraftment of 28 (IQR 22.5, 31.5) days and 26 (IQR 15, 34) days, respectively (48). In a recent report for children with non-malignant disease who received a TCRαβ+/CD19+ (αβT/CD19) depleted haplo-HSCT after reduced-toxicity myeloablative conditioning, consisting of treosulfan, fludarabine, and thiotepa or fludarabine, cyclophosphamide and a low dose of total-body irradiation, the median engraftment time for neutrophils and platelets were 14 and 12 days from graft infusion, respectively (49). Arnold et al. reported a median time to neutrophil recovery of 13 days in pediatric patients with hematologic malignancies who received αβT/CD19 depleted HCT from unrelated donors following myeloablative conditioning with busulfan or total body irradiation, cyclophosphamide and thiotepa (50). Similarly, a median time of 13 and 15 days for neutrophil and platelet engraftment was reported in a prospective multicenter phase I/II trial of adults and pediatric patients with malignant diseases and non-malignant disorders receiving αβT/CD19 depleted HCT from haploidentical family donors after a reduced-intensity conditioning with fludarabine, thiotepa, and melphalan (51). Bleakley et al. recently reported a T-cell depletion approach utilizing naïve (CD45RA) T-cell depletion in patients with malignancies, which resulted in rapid engraftment (52). Neutrophil engraftment occurred at a median of 15 (9-29) days, and platelet counts exceeded 20,000/mm3 without transfusion at a median of 14 (8-111) days (52). This method will need to be studied in this patient population but appears promising. Overall, our approach with CD34 selection and PB MNC addback was comparable to αβT/CD19 depletion and the naïve T-cell depletion approaches, and more robust than the T-replete post-cyclophosphamide method of haploidentical transplantation.

Our trial utilized grafts that were CD34 selected with a fixed T-cell add back (CD3 2X10^5^/kg). This methodology of graft selection is different than other published haploidentical transplant approaches for SCD. In our trial, the pace of CD4, CD56 and CD19 immune cell reconstitution was similar to that reported for the αβT/CD19-depleted grafts, however, the recovery of CD8 cells in our trial was significantly delayed compared to the αβT/CD19 depletion trial 60 days vs 270 days but similar at 213 days, and also similar to reconstitution in patients given naïve T-cell depleted grafts (28, 52). The recovery of CD19 cells in our trial was quicker compared to CD3/CD19 depletion 86 days vs 60 days (28), and similar to αβT/CD19 depleted grafts (46 days). Oostenbrink reported on 14 patients who received a mismatched MUD or haploidentical transplant for hemoglobinopathies (47). NK-cell recovery and B-cell recovery in the mismatched PTCy group was significantly slower compared to our approach (47). Our transplant regimen that we utilized in this clinical trial is very intensive and has contributed to the high degree of donor chimerism. Other clinical trials using haploidentical donors and post transplant cyclophosphamide have utilized anti-thymocyte globulin, fludarabine 150 mg/m^2^ cyclophosphamide 129 mg/kg with total body irradiation 400 cGy with T replete haploidentical donors with 6% primary graft failure and 18% had mixed donor-host chimerism (48). In a different study using haploidentical donors and alpha beta depleted grafts the investigators utilized a preconditioning phase, with hydroxyurea and azathioprine from day −59 pretransplant and fludarabine 180 mg/m2,busulfan (ablative), thiotepa, and cyclophosphamide (200 mg/kg) and ATG. This regimen had a 14% graft failure rate (46). The intensity of this regimen is very similar to the intensity of our own. However due to our successful engraftment and chimerism, we are investigating a de-escalation of our current conditioning regimen and decreasing the dose of Cytoxan by 50% with the ultimate goal of removal in subsequent studies. We utilize total lymphoid irradiation which is novel to our study in this patient population that may contribute to the high engraftment rates and sustained chimerism and fewer late effects than Total Body irradiation used with other regimens. We are monitoring closely for late effects in our survivors and collecting that additional data. To date we have not noticed serious toxicities and have seen improvement in lung function over time due to the correction of sickle cell disease. The only transplantation-related mortality related to the conditioning regimen that we found in our study was one patient who died of veno-occlusive disease. This is a common occurrence in SCD due to the iron overload from chronic transfusions.

Gaziev et al. reported on 14 children with hemoglobinopathies transplanted with αβT/CD19-depleted grafts or CD34 selected without an addback using a conditioning regimen with busulfan, thiotepa, cyclophosphamide, and antithymocyte globulin. CMV, EBV, ADV, and BKV reactivations were reported in 64%, 28%, 7%, and 23%, respectively (46). This compares to our cohort where CMV reactivations were 26%, EBV 26%, and ADV 10%. In a T replete haploidentical approach with PTCy, de La Fuenta reported asymptomatic viral reactivation, mainly CMV (20%; 3 of 15) and EBV (40%; 6 of 15), occurred in 15 patients (60%) by day +100 (37). Oostenbrink reported CMV reactivation took place in 6 of 11 at risk mismatched PT-CY patients (47). Thus the viral reactivation rate appears similar in both the T-cell depleted and PTCy approaches. Gaziev also noted 38% of patients developed bacterial sepsis. In our cohort there were 52% who developed bacterial infections. The high incidence of bacterial sepsis in SCD patients and strategies for continued antibacterial prophylaxis warrants further investigation. SCD patients have high iron burdens that cause the spleen to be nonfunctional due to chronic sickling, this added to defects in complement activation put them at risk for bacterial infections (53).

NK cells reconstitute earlier than T and B cells, generally within 30 days following allogeneic HSCT irrespective of the graft source (54). A high early (<8 weeks) recovery of allogeneic NK cells was previously reported to be associated with less aGVHD, reduced transplant-related mortality, fewer infections, and improved survival in cancer patients (54–57). As previously reported, we observed a rapid NK reconstitution that peaked at day 30 (**Figure 7A**). The rapid NK reconstitution may contribute to the excellent (90%) 1-year EFS (38) and low aGVHD (**Figure 2C**). Further analysis utilizing cox proportional hazards models, will be needed to investigate the associations.

Previous studies have shown that the rapid reconstitution of NK cell activating receptors benefits NK cell activity after HSCT (58). We analyzed NK cell receptor repertoire in the recipients before and after SCT at days 30, 60, 100 and 180. Earlier studies utilizing umbilical cord blood transplant, CD34 enriched grafts, or other HSCT showed that NK reconstitution was accompanied by an increase in the ratio of CD56^bright^ to CD56^dim^ NK cells and a high expression of CD94/NKG2A-inhibitory receptors (58–60). Similarly, we found that the ratio of CD56 bright to CD56 dim NK cells was higher at day 30 (**Figure 7A**). NKG2A and CD94 were significantly increased at 30 days after HSCT as compared to pre-transplantation (**Figure 7B**) (NKG2A: 33.2 ± 8.55% vs 3.53 ± 0.53%; CD94: 27.8 ± 9.32% vs 4.02 ± 1.09%). However, a sustained high number of NK cells that express CD56 bright and NKG2A/CD94 has often been associated with higher transplantation-related mortality and poorer leukemia-free survival (61). Importantly, consistent with the previous findings of NK receptors in CD3/CD19-depleted or CD34 enriched grafts, the expression of activating receptors (NKp44 and NKp46, KIR2DS and NKG2D) on donor NK cells (**Figure 7**) was rapidly reconstituted at day 30 and was maintained until day 180. This could override the inhibition from NKG2A/CD94 binding to its HLA-E ligand and allow NK cells to eliminate cancer cells or infected cells. In agreement with the expression of high levels of NK activating receptors, we observed enhanced NK cell cytolytic activity against K562 cells at day 30 over baseline (**Figure 7C**) with enhanced NK activating marker CD107a expression (**Figure 7D**) and enhanced degranulation propensity (**Figure 7E**). These important lines of evidence suggest that after HISCT, NK cells mature quickly resulting in rapid appearance of functional NK cells.

In summary, immune reconstitution and time to engraftment in our trial is very similar to the αβT/CD19 depletion and naïve T-cell depletion approaches (52) and shows benefit comparable to the T-replete post-cyclophosphamide approach. Since CD34 selection using the CliniMACS Plus device is FDA approved under a Humanitarian Use Device application, this method of T-cell depletion is accessible to more centers outside the context of a clinical trial and provides comparable results to αβT/CD19 depletion. Following CD34 enrichment and MNC addback, engraftment of functional NK cells occurred rapidly, which suggests that MNC addback facilitates ideal conditions for NK cell recovery. Future comparative cellular engineering strategies comparing methodologies will help identify the optimal approach.

## Data availability statement

The original contributions presented in the study are included in the article/**Supplementary Material**. Further inquiries can be directed to the corresponding author.

## Ethics statement

This multi-site phase II study was approved by the institutional review board at each participating site prior to enrollment. Each patient and/or guardian signed a written informed assent/consent. This study was registered at clinicaltrials.org (NCT02675959). Written informed consent to participate in this study was provided by the participants’ legal guardian/next of kin.

## Author contributions

MC, J-AT, YC, and LB-L participated in study design, data interpretation, and manuscript preparation. YC, LB-L, CK, JV, JA, BJ, and RW were responsible for laboratory investigation. QS, CV, and YC performed statistical data analyses. ER, HM TM, BG, SS, and SF collected the patient’s data used in this study. All authors reviewed and approved of the final draft of the manuscript prior to submission.
