# Supplementary material for: Donor chimerism and immune reconstitution following haploidentical transplantation in sickle cell disease
Source: Front Immunol. 2022 Dec 9;13:1055497. doi: 10.3389/fimmu.2022.1055497 (PMC9780682; doi:10.3389/fimmu.2022.1055497)
Supplement: Supplementary file 1 [file DataSheet_1.pdf]

Supplementary Material

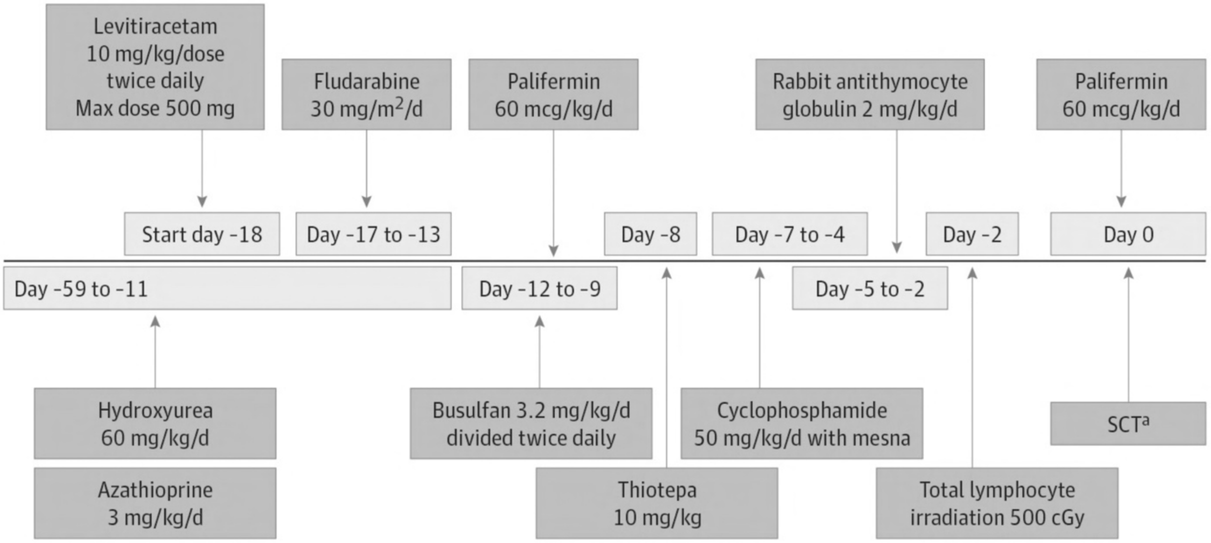

Supplementary Figure 1. Myeloimmunoablative conditioning regimen and supportive care. Patients who underwent stem cell transplant received hydroxyurea and azathioprine starting day –59 to day –11, fludarabine on days −17, −16, −15, −14, and −13; busulfan twice daily on days −12, −11, −10, and −9; thiotepa on day −8; cyclophosphamide on days −7, −6, −5, and −4; total lymphocyte irradiation on day −2; and rabbit antithymocyte globulin on day −5, −4, −3, and −2.

Reproduced with permission from: Cairo M.S., Talano J., et al. (2020), Familial Haploidentical Stem Cell Transplant in Children and Adolescents With High-Risk Sickle Cell Disease: A Phase 2 Clinical Trial. JAMA Pediatrics, 174(2):195-197. doi:10.1001/jamapediatrics.2019.4715

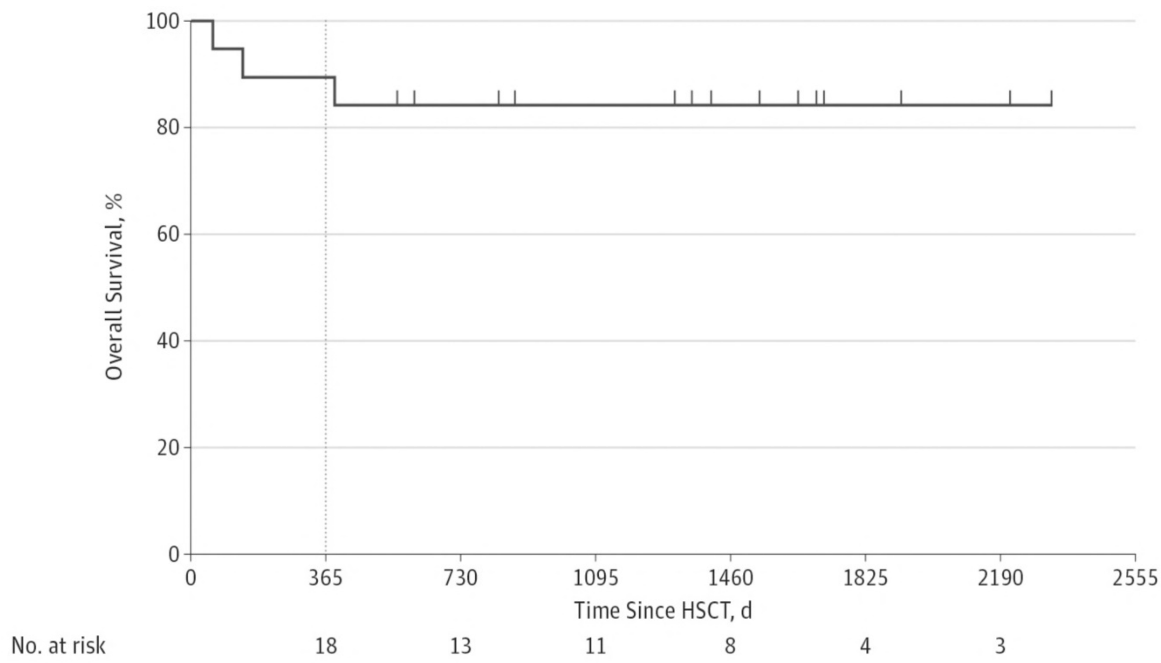

Supplementary Figure 2. Probability of overall survival. Probability of overall survival in 19 enrolled patients following familial haploidentical stem cell transplant using donor $CD34^+$ enrichment and mononuclear cell addback determined by the Kaplan-Meier product limit method. HSCT, haploidentical stem cell transplant; Max, maximum; SCT, stem cell transplant.

Reproduced with permission from: Cairo M.S., Talano J., et al. (2020), Familial Haploidentical Stem Cell Transplant in Children and Adolescents With High-Risk Sickle Cell Disease: A Phase 2 Clinical Trial. JAMA Pediatrics, 174(2):195-197. doi:10.1001/jamapediatrics.2019.4715

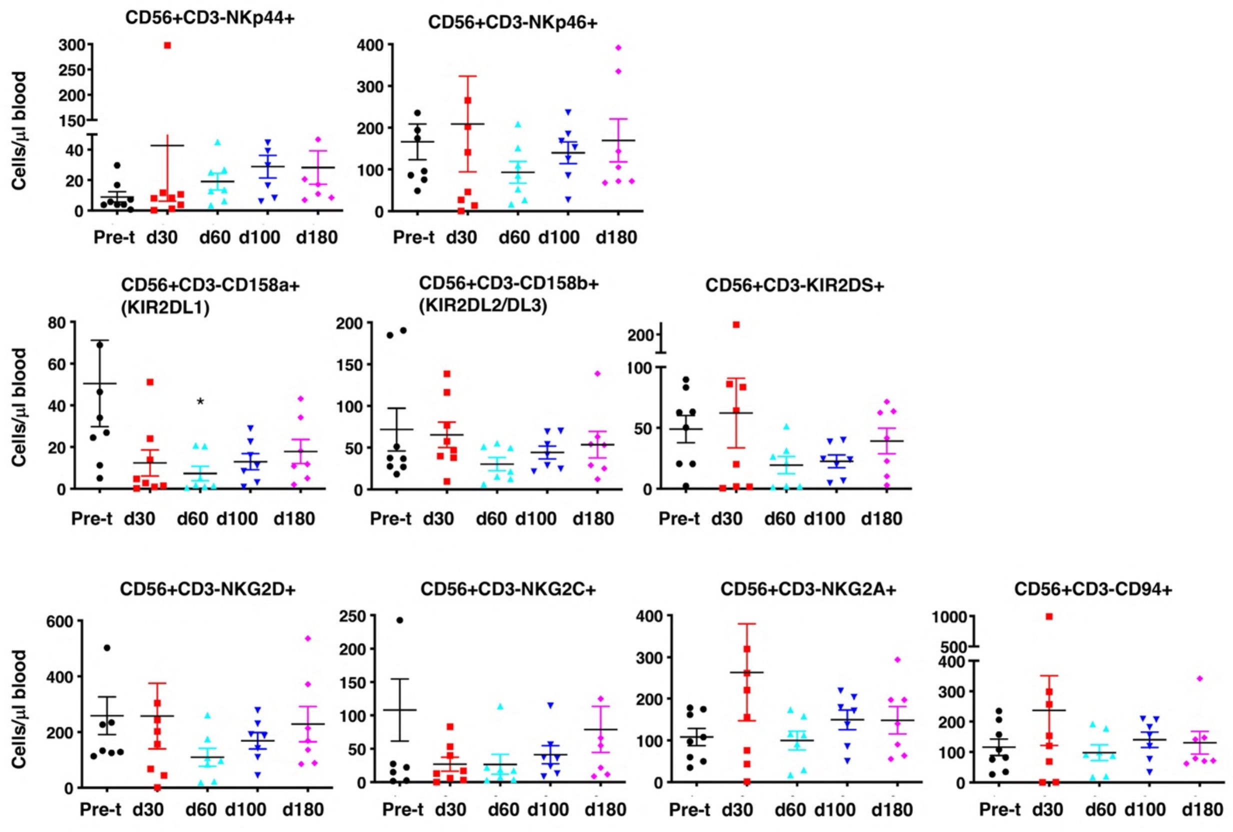

Supplementary Figure 3. Reconstitution and Function of NK cells and NK cell subsets. Absolute counts of NK cell subsets (NKp44, NKp46, CD158a, CD158b, KIR2DS, NKG2D, NKG2C, NKG2A and CD94) were measured before and after HISCT at days 30, 60, 100, and 180. HISCT, haploidentical stem cell transplantation.
